# A Symmetric Three Degree of Freedom Tensegrity Mechanism with Dual Operation Modes for Robot Actuation

**DOI:** 10.3390/biomimetics6020030

**Published:** 2021-05-18

**Authors:** Tianyuan Wang, Mark A. Post

**Affiliations:** Department of Electronic Engineering, University of York, Heslington, York YO10 5DD, UK; tw1356@york.ac.uk

**Keywords:** tensegrity, robot, modular, bio-inspired, simulation, compliance

## Abstract

Tensegrity robots that use bio-inspired structures have many superior properties over conventional robots with regard to strength, weight, compliance and robustness, which are indispensable to planetary exploration and harsh environment applications. Existing research has presented various tensegrity robots with abundant capabilities in broad scenarios but mostly not focused on articulation and manipulability. In this paper, we propose a novel tensegrity mechanism for robot actuation which greatly improves the agility and efficiency compared with existing ones. The design integrates two separate tensegrity substructures inspired by shoulder and hip joints of the human body and features a similar form to a hexapod platform. It mitigates detrimental antagonistic forces in the structural network for optimising actuation controllability and efficiency. We validated the design both on a prototype and in a Chrono Engine simulation that represents the first physically accurate simulation of a wheeled tensegrity robot. It can reach up to approximately $58.9^{\circ }$, $59.4^{\circ }$ and $47.1^{\circ }$ in pitch, yaw and roll motion, respectively. The mechanism demonstrates good agility and controllability as an actuated robot linkage while preserving desirable properties of tensegrity structures. The design would potentially inspire more possibilities of agile tensegrity implementations that enable future robots with enhanced compliance, robustness and efficiency without a tradeoff.

## 1. Introduction

Research interests for planetary exploration and harsh environment operation have been a long-term focus in robots. Due to terrain irregularity, the presence of environmental physical hazards and the need for rocket-based transport, it is challenging to sustain robot durability and reliability. These lead to robots for these applications having high demands in robustness, survivability, traversing ability and light weight, similar to demands on many kinds of biological systems.

Conventional rigid body robots perform well in many aspects such as precision and manipulability, but tensegrity robots are more adaptable and flexible, similar to animals or plants in physical capabilities and responses, and they can be competitive for the above applications due to the features of the tensegrity structure. In rigid robots, the wide use of leverage linkages and orthogonal attached components result in a large mass, as a tradeoff for building stiffness and strength [1]. Additionally, single point mechanical failures, which easily occur due to abrasion or external impact, normally lead to total failure of the linkage and severely affect the performance of the whole robot.

Tensegrity structures are derived from observations of geometric principles and structures in nature and are naturally compliant to external forces while retaining a measure of stiffness. The structure expands to fill space in a non-intuitive manner. It is composed of discontinuous rigid elements in compression and a continuous network of elastic elements that are in tension [2]. Such a spatial arrangement of elements gives tensegrity structures several common characteristics with biological structures such as stability, structural efficiency, reconfigurability, failure tolerance, deployability and ease of modelling [3,4]. Emerging studies have been carried out on tensegrity robots exploiting these advantages since the 2010s. These robots adopt a wide range of morphologies including the icosahedron [5], spine [6,7], prism [8], worm [9], tetrapod [10], spherical [11], quadruped [12,13], etc., Many tensegrity spine structures in particular are modelled on human spine structural shapes and characteristics and are controllable using bio-inspired strategies such as the Central Pattern Generator [14]. These former four listed tensegrity characteristics are well correlated to the demands of space robotics proposed above. A representative implementation for planetary exploration is the SUPERball series robots designed by NASA which realise high impact resistance and continuous rolling locomotion of a tensegrity icosahedron [15].

In this paper, we propose a novel tensegrity mechanism that builds on the principles of bio-inspired tensegrity targeting the demands mentioned above for robot actuation purposes in such scenarios, which is composed of two co-axially and symmetrically arranged tensegrity substructures. The mechanism achieves both superior motion efficiency and structure efficiency instead of a tradeoff in existing designs, and as a result it improves the agility leading to better actuation performance in practice. We also simulated the mechanism on the Chrono Engine physics simulator and validated the design with a prototype wheeled robot. This is the first time that a wheeled tensegrity robot has been simulated using a physically-accurate simulator for both tensegrity forces and wheeled movement. The key features of the mechanism include:

- A robust and light weight linkage: The mechanism is mass efficient as a linkage to stably connect two bodies of a robot while providing fault tolerance of its elements, making it suited for planetary exploration, mass sensitive scenarios and applications in harsh environments.
- Dual mode operation: The mechanism can operate fully passive or actuated, without losing its structural integrity. This enables the robot to adapt to different scenarios to save energy when necessary.
- Good manipulability: The mechanism is agile and easy to control compared with existing tensegrity implementations. It is suitable for applications requiring a large degree of deformation and accurate estimation of state.
- Easy combination with conventional robot frameworks: The mechanism realises a clean transition between tensegrity and conventional structures, enabling a rapid attachment and detachment to target robots, and with only minor interference with the payload of the conventional segment.

The outline of the paper is organised as follows. The design concept of what and how existing issues of tensegrity robots that our mechanism targets on are firstly presented. Then, the geometric model of the proposed tensegrity mechanism is analysed to estimate force composition and determine its theoretical workspace. The method of how the mechanism is simulated and practically tested is then introduced. In the following sections, the data of both simulation and prototype tests are extracted and analysed to validate each other and the design, as well as evaluate the properties of the mechanism. The conclusions and future work are discussed at the paper’s end.

## 2. Materials and Methods

### 2.1. Design Concept

Although tensegrity robots demonstrate decent capability in many aspects of mechanical application, they are normally not motion efficient, i.e., either are slow in actuation or locomotion or require excessive forces to be actuated. This is due to the force distribution property of tensegrities which leads to multiple antagonistic forces present across the whole network for any state change in a single element and eventually results in degraded manipulability. In addition, as tensegrity elements are scattered across a large 3D space, the structure is more complex than conventional ones. This causes the issue of increased complexity and computation for control of the structure as well as installation of payload. In short, it is crucial to improve manipulability, power efficiency and load capacity for tensegrity robots, while retain desirable properties of the tensegrity structure.

The above shortcomings are most often observed in robots based on traditional Class-1 tensegrity structures, with compression elements made up of straight struts only. Focusing on these challenges, extended studies have investigated atypical tensegrity structures, such as bio-inspired, namely bio-tensegrity, that is the compression elements take various biological forms of skeletons or tension elements that mimic the operating principles of muscles, tendons or ligaments, as well as mechanism that can couple to conventional structures, to improve actuation of robots, such as wheeled tensegrity robots with compliant bodies [16]. Other recent designs that focus on specific tensegrity mechanisms have improved the manipulability and agility to a certain extent but did not address these issues completely. Lessard et al. presented a bio-inspired tensegrity elbow and shoulder that make use of antagonistic force pairs to realise ease of active control with 2 DoFs for each joint [17,18]. The HEDRA by Ramadoss et al. is a manipulator based on stacked tensegrity tetrahedrons which exploits a three-cable actuation scheme [19]. These two designs mitigate the detrimental antagonistic forces, but the structure’s failure tolerance is decreased. The design of Naribole et al. introduces extra cables to externally actuate a tensegrity tetrapod spine [20]. It improves control complexity of such form of tensegrity, but the antagonistic tension forces of its passive cables drain extra actuation power. Friesen et al. developed an agile joint which manipulates a tensegrity tetrahedron with 3 DoFs [21]. It is an approach to a versatile solution but the workspace is limited. Therefore, to further improve a tensegrity design, we introduced several key factors during development:

- Minimal detrimental antagonistic forces within the structure;
- Minimal intrusion of tensegrity structure into the conventional structure;
- Clean segmentation between tensegrity and conventional structure;
- Three degrees of rotational freedom; and
- Optimised power efficiency.

**Figure 1:**
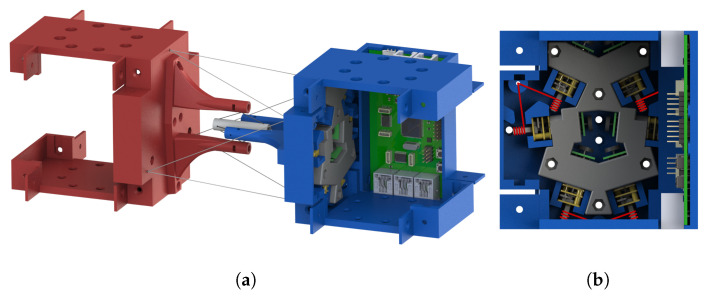
An overview of our proposed tensegrity mechanism at reference posture: only essential module tiles are assembled in the rendering; Electric wires connecting the PCB and the motors are not presented. (**a**) The 3D view of the whole mechanism. (**b**) The cross section side view along the axial direction of the mechanism; cables wound around the motor shafts are illustrated by red lines.

**Figure 2:**
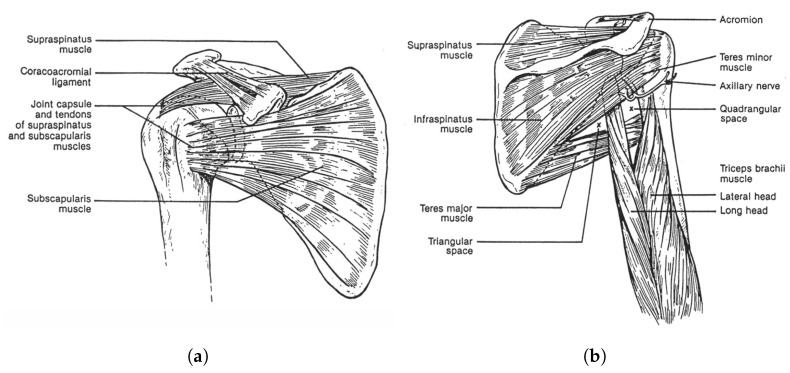
The shoulder anatomy of the shoulder joint and the rotator cuff muscles: the biological inspiration paradigm of the tensegrity mechanism. (**a**) The anterior view [22]. (**b**) The posterior view [22].

Figure 1 demonstrates a rendered overview of our proposed tensegrity mechanism. The agility of the mechanism is prioritised. We therefore took inspiration from the shoulder and hip joints of human body, the tensegrity structures in nature with 3 DoFs. We firstly furthered the arrangement of rigid universal joints in the form of tensegrity as the inner substructure, which yielded an extra DoF of roll motion and realised an approximate function of the shoulder or hip’s ball-and-socket joint. Figure 2 presents the anatomy of the shoulder joint and the rotator cuff muscles. The ball-and-socket joint is surrounded and primarily actuated by the supraspinatus, infraspinatus, teres minor and subscapularis muscles, which can be observed in the anterior and posterior views, as well as other muscles. These muscles work in pairs of flexors and extensors to pull the humerus for various movements. Learning from how the shoulder joint is actuated with agility, we added outer cables arranged in both parallel and intersection to actuate the inner substructure’s 3 DoFs, where, in this case, the inner substructure act as the shoulder joint and the outer cables act as the muscle group. Instead of inserting the force bearing points to the bones, that is, the struts of the mechanism, we inserted the points to a surface perpendicular to the struts being pulled to reduce the force needed on the cables. We employed a full actuation approach on all six outer cables so that they work in beneficial antagonistic pairs similar to the flexors and extensors. This arrangement eventually resulted in a morphology similar to parallel manipulators and eliminated detrimental antagonistic forces in the structure, for both the inner and outer substructures, and greatly improved the motion efficiency. Such configuration uses cables extensively. The whole mechanism features a cable to strut length ratio of approximate 38:1 at the reference posture, leading to a high mass efficiency.

To improve the compatibility of the mechanism with conventional bodies in a robot, we cleanly separated the structural and mechanical parts of the mechanism. In typical tensegrity robots, the vertices or nodes normally scatter irregularly in 3D space, making it hard fit them well to conventional robots, and normally requiring a mounting face of large area. In addition, such a morphology limits the installation of the actuators so that the motors and electronics commonly have limited space to be attached to the struts. Unlike typical tensegrities, the vertices on each end of our mechanism lie on a flat face. Therefore, the tensegrity structure can be easily attached to conventional robots, and the mechanical and electronic parts can be contained in the rigid body without extra loads fitted on the structural parts or exposed to the environment. Only cable routing through holes are reserved on the faces for actuation. As a result, the mechanism could be applied for robot actuation as a conventional manner but introduce advantages of tensegrity to the system. In addition, we made the inner substructure independently functional. This further improves its motion efficiency for operation when outer cables are in a loose condition, i.e., a dual mode operation.

### 2.2. Numerical Analysis

**Figure 3:**
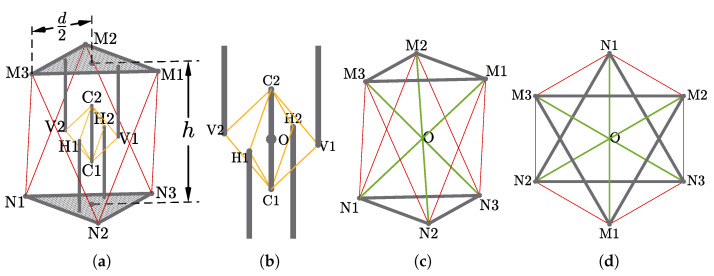
Geometric Models of the Mechanism: (**a**) The full view of the model where cables are represented by red and yellow lines rigid elements are represented by grey solids; (**b**) the passive substructure, composed of passive cables (yellow lines) and struts (grey bars); and (**c**,**d**) the active substructure’s 3D and top view where the passive substructure is simplified, composed of actuated cables (red lines) and bases (grey triangles).

The abstracted geometric model of the tensegrity mechanism is presented in Figure 3a. Its exterior surfaces form an octahedron of which the top and bottom faces are equilateral triangles. The whole mechanism is divided into two individual parts, the inner passive substructure and the outer active substructure. The role of the inner substructure, represented by the yellow lines and grey bars, is to retain the integrity of the tensegrity mechanism. The outer substructure, represented by the red lines and grey triangles, provides external actuation to the mechanism. Based on a separated substructure design, the mechanism can remain its structural integrity without presence of the active cables. The whole mechanism consists of 3 rigid elements and 14 cables without concatenated stages, considerably reducing the complexity of modelling. The overall height *h* is equal to the diameter *d* of circumscribed circle of the top/bottom faces, which is compatible with the specifications of the Sub-Modular Cube proposed by Post and Austin [23].

#### 2.2.1. Universal Joint-Like Passive Substructure

The inner passive substructure (Figure 3b) consists of 8 cables, arranged as a group of 4 on 2 orthogonal planes, and 5 struts, of which 4 struts are fixed to the top and bottom faces and 1 strut is suspended in the centre of the whole structure. The passive substructure acts as a universal joint while takes the form of tensegrity and introduces extra rotational freedom about axial axis of the central strut.

Since there is no constraint on the normal direction of the $H_{1}C_{1}H_{2}C_{2}$ and $V_{1}C_{1}V_{2}C_{2}$ planes of the passive substructure at reference posture, it is critical to minimise potential displacement along such directions. Take cable $H_{1}C_{1}$ as an example; assuming an equivalent tension force for all candidate cable rest lengths and a small displacement along the normal of the plane $H_{1}C_{1}H_{2}C_{2}$, the force $F_{d}$ pulls the central strut to the neutral position provided by this cable can be given by:

(1) $$F_{d}=\frac{EA}{L}\cdot \left(\Delta l_{0}+\Delta l_{d}\right)\cdot cos(\theta^{\prime })\cdot sin(\alpha )\approx \frac{EA}{L}\cdot \Delta l_{0}\cdot cos(\theta )\cdot sin(\alpha ),$$

where $\Delta l_{0}$ and $\Delta l_{d}$ are the length displacements of the cable $H_{1}C_{1}$ introduced by the original pretension and the small normal displacement, respectively; θ is the angle between $H_{1}O$ and $H_{1}C_{1}$; $\theta^{\prime }$ is the new θ resulted by the deviation; α is the angle of $H_{1}O$ deviating from its neutral position; and $\frac{EA}{L}\Delta l_{0}$ is a constant.

Meanwhile, the passive substructure should tend to retain its position for displacements along axial axis of the central strut. The displacement $\Delta x_{a}$ for an axially applied force $F_{a}$ can be given by:

(2) $$\Delta x_{a}=\frac{F_{a}\cdot L}{EA\cdot sin(\theta )}=\frac{F_{a}\cdot r_{p}}{EA\cdot sin(\theta )\cdot cos(\theta )},$$

where $r_{p}$ is the constant length of $H_{1}O$.

For the former case, $F_{d}$ should achieve the maximum value for any given pretension force, while $\Delta x_{a}$ seeks for the minimum value. Therefore, to determine the optimal value of θ, the equation for the derivatives of both cases with respect to θ is constructed as follows:

(3) $$-EA\cdot \frac{\Delta l}{L}\cdot sin\left(\alpha \right)\cdot sin\left(\theta \right)=\frac{F_{a}\cdot r_{p}}{EA}\cdot \left(\frac{1}{cos^{2}\left(\theta \right)}-\frac{1}{sin^{2}\left(\theta \right)}\right),$$

Since sin(α) is negligible, the optimal point is the intersection between the right hand side and the *x* axis, which results in $\theta =\frac{\pi }{4}$.

#### 2.2.2. Parallel Manipulator-Like Actuated Connection

The outer active substructure is composed of 6 individually actuated cables that connecting the top and bottom faces. The topology is similar to a parallel manipulator or a hexapod platform, but rotates about the origin *O*, providing actuated 3 degrees of rotational freedom with no translational freedom. A simplified model is demonstrated in Figure 3c,d.

At reference posture, faces $N_{1}N_{2}N_{3}$ and $M_{1}M_{2}M_{3}$ are $180^{\circ }$ rotated leading to a symmetrical arrangement of the structure. Assume the face $N_{1}N_{2}N_{3}$ is fixed, the coordinates of vertices of face $M_{1}M_{2}M_{3}$ can be given by:

(4) $$\begin{array}{cc} m_{i}^{{}^{\prime \prime \prime }} & =R_{yXZ}m_{i}\\ \; & =\left[\begin{array}{ccc} c_{1}c_{3}+s_{1}s_{2}s_{3} & c_{3}s_{1}s_{2}-c_{1}s_{3} & c_{2}s_{1}\\ c_{2}s_{3} & c_{2}c_{3} & -s_{2}\\ c_{1}s_{2}s_{3}-c_{3}s_{1} & c_{1}c_{3}s_{2}+s1s3 & c_{1}c2 \end{array}\right]m_{i},\;i=1,2,3 \end{array}$$

with

$$R_{yXZ}=R_{zxy}=R_{y}\left(\alpha \right)R_{x}\left(\beta \right)R_{z}\left(\gamma \right),$$

$$\begin{array}{cc} R_{y}\left(\alpha \right) & =\left[\begin{array}{ccc} cos(\alpha ) & 0 & sin(\alpha )\\ 0 & 1 & 0\\ -sin(\alpha ) & 0 & cos(\alpha ) \end{array}\right],\\ R_{x}\left(\beta \right) & =\left[\begin{array}{ccc} 1 & 0 & 0\\ 0 & cos(\beta ) & -sin(\beta )\\ 0 & sin(\beta ) & cos(\beta ) \end{array}\right],\\ R_{z}\left(\gamma \right) & =\left[\begin{array}{ccc} cos(\gamma ) & -sin(\gamma ) & 0\\ sin(\gamma ) & cos(\gamma ) & 0\\ 0 & 0 & 1 \end{array}\right],\\ m_{i} & =\left[\begin{array}{c} \frac{d}{2}cos\left(\frac{2\pi }{3}\left(i-1\right)+\frac{5\pi }{6}\right)\\ \frac{d}{2}sin\left(\frac{2\pi }{3}\left(i-1\right)+\frac{5\pi }{6}\right)\\ \frac{l}{2} \end{array}\right], \end{array}$$

where α, β and γ are Euler angles (Tait–Bryan convention) following the *Y*–*X*–*Z* rotation sequence, respectively; $m_{i}$ is the coordinate of top face vertices $M_{1}$, $M_{2}$ and $M_{3}$ at reference frame; and $m_{i}^{{}^{\prime \prime \prime }}$ is the coordinate of these vertices after the third Euler rotation.

#### 2.2.3. Workspace of the Tensegrity Mechanism

As the tensegrity mechanism can operate either passively or actuated, its workspace analysis is also divided into two domains. As for the passive mode, its maximum tilt and roll angels are mainly limited by mechanical constraints. Assuming the circumscribed circle of the top/bottom faces to be the base for simplicity, as the mechanism overall height *h* is equal to the diameter *d* of the base, it is clear that the mechanism yaw and pitch range is $\pm 90^{\circ }$. The roll range is $\pm 90^{\circ }$ as well due to the orthogonal placement of the struts.

To identify the mechanism’s actuation workspace, we used the Force Closure Check (FCC) algorithm proposed by Pham et al. [24], which is capable of checking high dimensional force-closure condition of a cable-driven parallel mechanism (CDPM). Assuming the cables can provide infinite tension forces, a specific posture of the tensegrity mechanism is considered within the workspace when it can form an equilibrium with internal and external applied forces and torques.

To determine the force-closure condition of a given posture, a structure matrix is firstly created to describe force and torque conditions of the mechanism. Since our mechanism does not have translational freedom, the structure matrix *A* can therefore be given by:

(5) $$A=\left[m_{1}^{{}^{\prime \prime \prime }}\;\times \;u_{11}\;m_{2}^{{}^{\prime \prime \prime }}\;\times \;u_{21}\;m_{2}^{{}^{\prime \prime \prime }}\;\times \;u_{22}\;m_{3}^{{}^{\prime \prime \prime }}\;\times \;u_{32}\;m_{3}^{{}^{\prime \prime \prime }}\;\times \;u_{33}\;m_{1}^{{}^{\prime \prime \prime }}\;\times \;u_{13}\right],$$

with

$$u_{ij}=\frac{n_{j}-m_{i}^{{}^{\prime \prime \prime }}}{|n_{j}-m_{i}^{{}^{\prime \prime \prime }}|},\;\;ij\in \left\{11,21,22,32,33,13\right\}$$

$$n_{j}=\left[\begin{array}{c} \frac{d}{2}cos\left(\frac{2\pi }{3}\left(j-1\right)-\frac{5\pi }{6}\right)\\ \frac{d}{2}sin\left(\frac{2\pi }{3}\left(j-1\right)-\frac{5\pi }{6}\right)\\ -\frac{l}{2} \end{array}\right],$$

where $u_{ij}$ is the unit vector representing the force direction applied on vertex $m_{i}^{{}^{\prime \prime \prime }}$, that is collinear with the active cables, and *A* is actually a 3-by-6 torque matrix of which each column elements representing a torque provided by a cable.

Then, for each torque vector in the matrix, the other torque vectors are projected onto the hyper plane orthogonal to this vector to form a lower dimension matrix filled with new non-zero torque vectors, recursively, until the column dimension is 1. Through this process, the structure matrix *A* is reduced twice on its column dimension to generate 6 2-by-5 matrices and 6×5 1-by-4 matrices. For each row in all generated matrices, the sign of the elements is checked. Whenever there is an all negative or all positive row, the posture under inspection fails with FCC.

### 2.3. Simulation Methods

**Figure 4:**
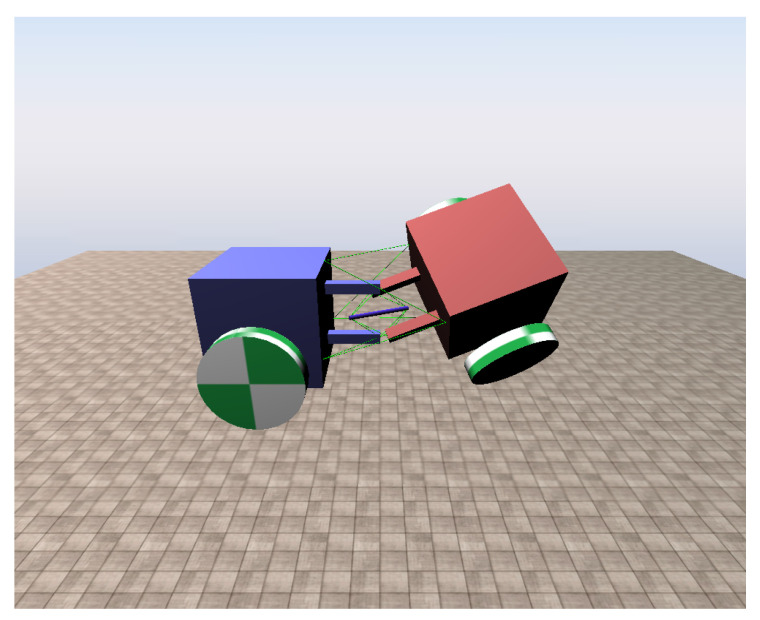
Rendering of the tensegrity mechanism simulation scene in a random posture.

The simulation of our tensegrity mechanism was implemented on Chrono Engine, an open source multi-physics dynamics engine with validated fidelity [25,26]. For existing studies, the NASA Tensegrity Robotics Toolkit (NTRT) simulator is a commonly used platform [27]. However, it is developed for tensegrity structures only, which means it cannot simulate tensegrity robots containing conventional links or robotic elements such as wheels as a complete system. One of the contributions that our simulation makes is that it enables an integrated simulation of tensegrity structure and conventional rigid body robots in one scene. The cables of our simulation are modelled with Finite Element Method (FEM), and Chrono Engine can take material data as input parameters for FEM as well as other elements. Thus, the simulation objects can be modelled matching real world objects more easily and honestly. A rendering of the tensegrity mechanism simulation scene is presented in Figure 4. The coordinate system of the simulation is based on the Vehicle module of the engine, where in the figure *z* axis points upwards, *x* axis points leftwards and *y* axis points out of the paper.

#### 2.3.1. FEM Cable Based Simulation

For the end effectors and struts of the mechanism, we used rigid “ChBody” elements with ABS material parameters, an average Young’s modulus of 2.3 GPa and a density of 1.15 g/cm${}^{3}$. For the experiment presented in this paper, we focused on the evaluation of the tensegrity mechanism. We therefore fixed one of the end effector to the world and made the other one free of movement in the air, and set the mass of the wheels to be negligible. The overall mass of each end effector is about 173 g. Since there are no collisions or contacts of rigid elements, the static and sliding friction coefficients were left at the default value of 0.6. The cables were created with the “ChElementCableANCF” elements, which is based on the Absolute Nodal Coordinate Formulation (ANCF) that has been validated according to the technical report [28]. The cables were configured to match Nylon material with a diameter of 0.5 mm. The active cables share a common pretension ratio and the passive cables pretensioned twice that of the active ones. The gravity was set to 9.8 m/s${}^{2}$ with the mechanism horizontally placed to evaluated the resulted effect.

#### 2.3.2. Quaternion Based Motion Control

Following the analysis convention in Section 2.2, we used yXZ sequence Euler angles to represent target posture in the simulation. Owing to the simplicity of the structure, the inverse kinematics can be easily derived from the target rotation angles using $l_{ij}=\left|n_{j}-m_{i}^{{}^{\prime \prime \prime }}\right|$ and Equation (4) to control the mechanism. However, repeated multiplication of successive rotation matrices during simulation requires intensive computation. We therefore used quaternions to calculate the coordinate of the vertices to improve efficiency. The conversion from Euler angles to quaternions is given by:

(6) $$\begin{array}{cc} q & ={\left[q_{w}\;q_{x}\;q_{y}\;q_{z}\right]}^{T}\\ \; & =\left[\begin{array}{c} cos\left(\frac{\gamma }{2}\right)cos\left(\frac{\alpha }{2}\right)cos\left(\frac{\beta }{2}\right)+sin\left(\frac{\gamma }{2}\right)sin\left(\frac{\alpha }{2}\right)sin\left(\frac{\beta }{2}\right)\\ sin\left(\frac{\gamma }{2}\right)cos\left(\frac{\alpha }{2}\right)cos\left(\frac{\beta }{2}\right)-cos\left(\frac{\gamma }{2}\right)sin\left(\frac{\alpha }{2}\right)sin\left(\frac{\beta }{2}\right)\\ cos\left(\frac{\gamma }{2}\right)sin\left(\frac{\alpha }{2}\right)cos\left(\frac{\beta }{2}\right)+sin\left(\frac{\gamma }{2}\right)cos\left(\frac{\alpha }{2}\right)sin\left(\frac{\beta }{2}\right)\\ cos\left(\frac{\gamma }{2}\right)cos\left(\frac{\alpha }{2}\right)sin\left(\frac{\beta }{2}\right)-sin\left(\frac{\gamma }{2}\right)sin\left(\frac{\alpha }{2}\right)cos\left(\frac{\beta }{2}\right) \end{array}\right], \end{array}$$

### 2.4. Prototype Configuration

The prototype of the mechanism features a small form factor. The structural part’s height *h* and base width *d* are both 100 mm that is consistent with simulation settings. The active cables are wound on motor shafts to control the rest length. The end effectors and struts are 3D printed with ABS filament and the cables are Nylon fishing threads. The motors are Pimoroni COM0801 298:1 Micro Metal Gearmotors mounted with 12 CPR magnetic encoders, driven by TB6612FNG motor drivers and an STM32F417VG micro controller. Due to the small form factor of the prototype, force sensors were not introduced to the system and the reference posture setup of the prototype was based on measurement of cables. With a wall thickness of 2 mm, the default active cable length is 110.9234 mm, and the overall mass of the free of movement end effector is about 172 g.

## 3. Results

### 3.1. Numerical Analysis Results

**Figure 5:**
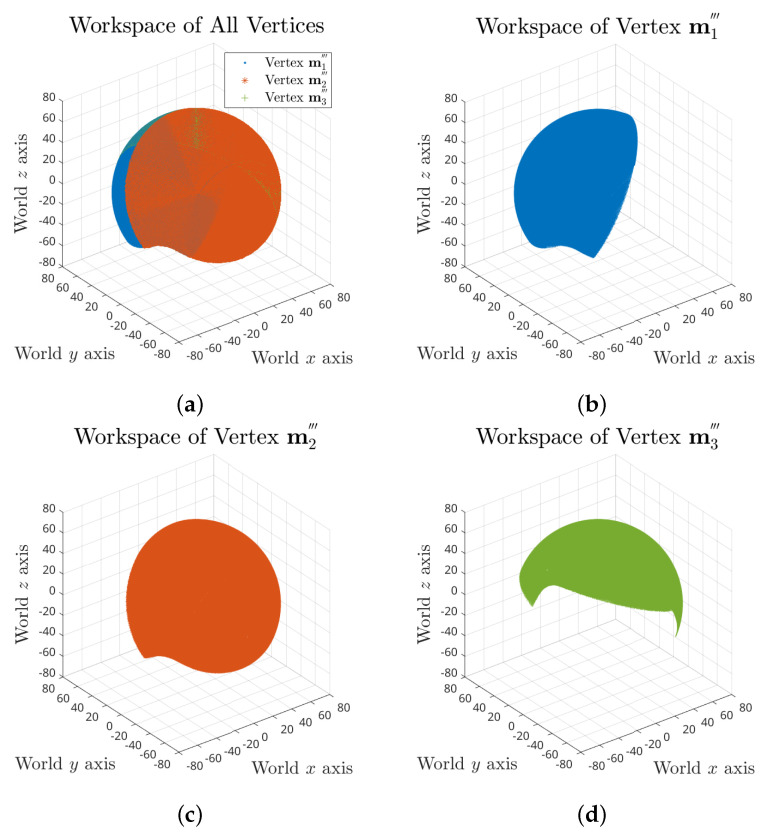
Full workspace of the tensegrity mechanism: (**a**) combined workspace plot of all three vertices; and (**b**–**d**) separate workspace plots of vertex $m_{1}^{{}^{\prime \prime \prime }}$, $m_{2}^{{}^{\prime \prime \prime }}$ and $m_{3}^{{}^{\prime \prime \prime }}$.

For the whole workspace, we took search trials on yaw, pitch and roll angle combinations of range $⟦-90^{\circ },90^{\circ }⟧$ for each, which results in a total number of 5,929,741 FCCs. The full workspace of the tensegrity mechanism is demonstrated in Figure 5, which consists of 2,141,901 valid postures.

**Figure 6:**
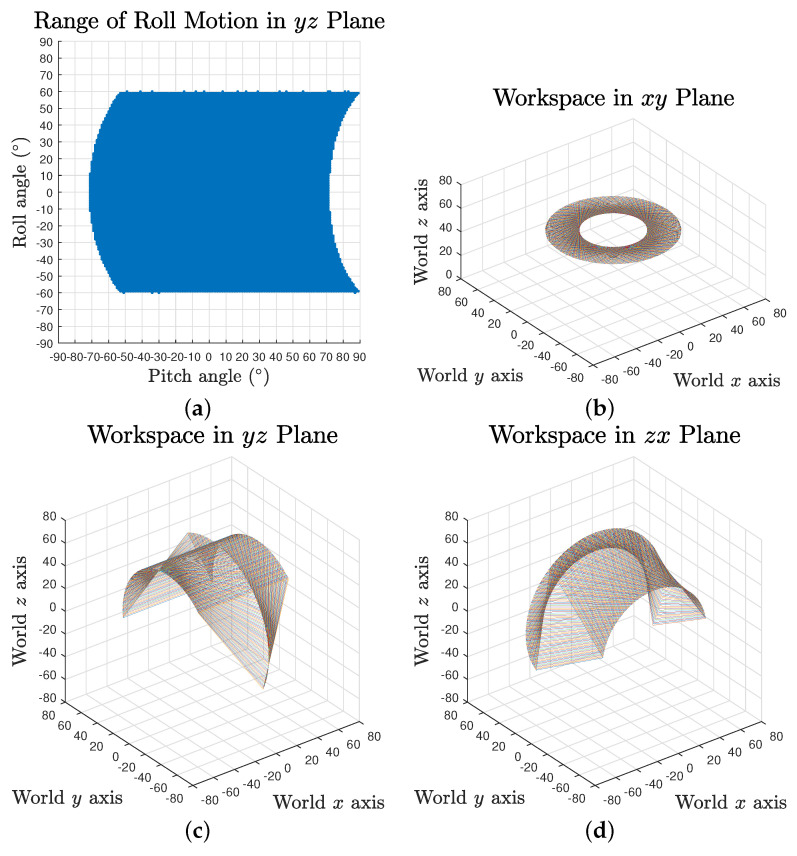
Workspace of the tensegrity mechanism in coordinate planes: (**a**) roll motion range in yz plane; and (**b**–**d**) roll motion in xy plane, pitch motion in yz plane and yaw motion in zx plane.

The full workspace result reveals that the maximum range of each rotation motion is variant and dependent on the values of the other two. Taking roll range output based on $0^{\circ }$ yaw angle and $⟦-90^{\circ },90^{\circ }⟧$ pitch angles as an example, the maximum swing decreases when the pitch angle is approaching the negative limit, as shown in Figure 6a. It can also be observed that the pitch motion could reach further in positive direction for some specific roll angles.

To provide an intuitive estimation of the workspace, the absolute actuation ranges for independent yaw, pitch and roll rotation were investigated, where the results are $\left[-81^{\circ },81^{\circ }\right]$, $\left[-71^{\circ },71^{\circ }\right]$ and $\left[-59^{\circ },59^{\circ }\right]$, respectively, as visualised in Figure 6b–d.

The FCC algorithm determines the theoretical maximum limits of the workspace. It uses the assumption of no extra mechanical constraint introduced to the system, such as the physical volume of the compression elements and the cable physical maximum tension force, as well as the effect of pretension of the system, which refers to the reality gap issues. Thus, the practical workspace is an implementation-dependent range varying from actual design to design but smaller than these absolute extremes.

### 3.2. Simulation Results

**Figure 7:**
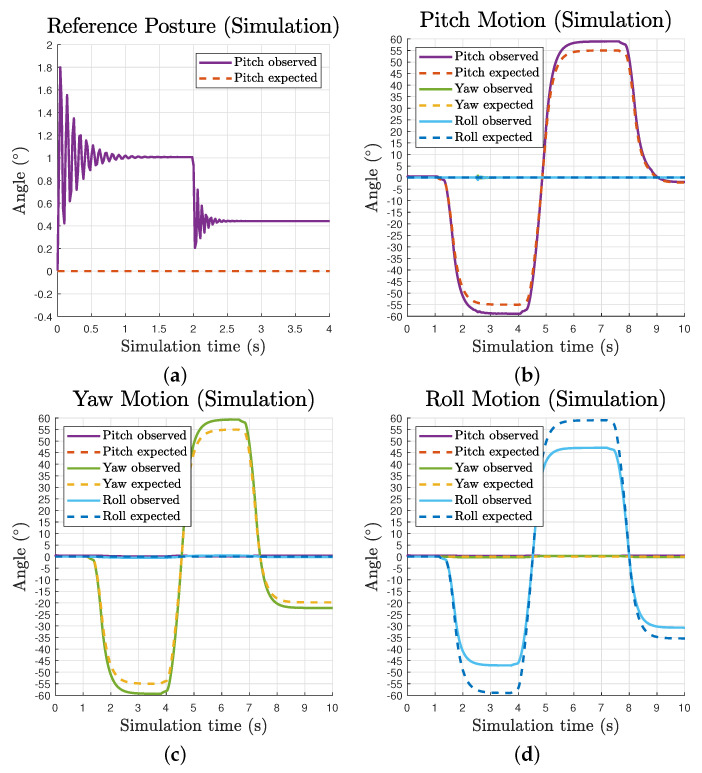
Waveforms of observed and expected motion of the movable end effector over simulation time: (**a**) reference posture showing oscillation and static deviation; and (**b**–**d**) pitch, yaw and roll individual motions.

Due to the volume of the passive substructure in simulation, we restrained the maximum pitch and yaw angles to $55^{\circ }$ to avoid cable collisions. The observed and expected motion data comparison of the movable end effector is presented in Figure 7b–d. With a uniform 95% pretension ratio, it is clear the observed motion waveforms follow expected lines for all three components motions. The maximum observed range of pitch, yaw and roll motion are approximately $\left[-59.0^{\circ },58.9^{\circ }\right]$, $\left[-59.4^{\circ },59.4^{\circ }\right]$ and $\left[-47.1^{\circ },47.1^{\circ }\right]$, respectively. It should be noted that there is a continuous deviation between the observed and expected line, and it increases over the growth of angle values. This is caused by the invariant uniform pretension ratio in all cables while the moment arms are continuously changing along operation. For pitch and yaw motions, the observed lines go beyond the inputs while the observed roll motion does not reach the input.

The pitch motion waveform at the reference posture reveals the inherent compliance of the tensegrity mechanism, as presented in Figure 7a. With the Earth surface gravity and a pretension ratio of 95%, the mechanism gives a static pitch deviation of about $0.4^{\circ }$ due to its self weight, while this value is about 2.5 times larger without pretension. The oscillation during motion caused by flexible tension elements in the system is also perceivable as well as the settling time difference affected by changes in tension force.

**Figure 8:**
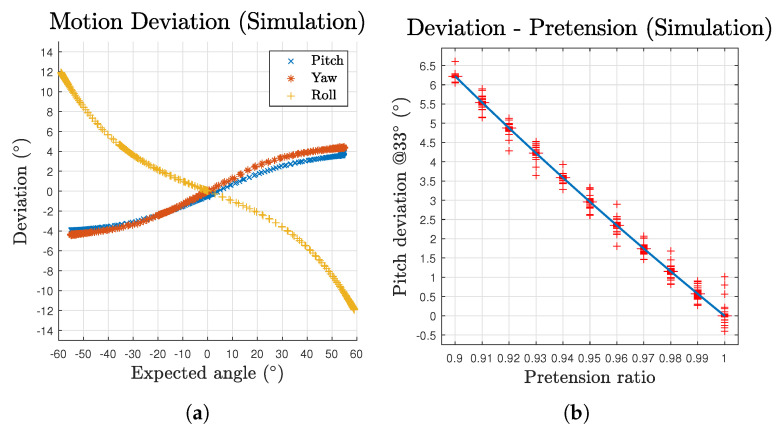
Deviation of the mechanism’s motion in simulation: (**a**) deviation with respect to expected angles for all three motions; and (**b**) deviation with different pretension ratios at an expected pitch angle of 33°.

The deviation to motion angle relationships are plotted in Figure 8a. The scatters are composed of both outbound and inbound for the two directions of the three component motions. Each of the three motions’ scatters generally form one single line, demonstrating consistent performance of the mechanism in space domain. According to the deviation results, although the deviation increases over the growth of motion angle, the pitch and yaw motions’ growth curves gradually slow down. In addition, the effect of gravity is perceivable over the whole pitch motion workspace as a persistent offset on its curve, making the curve not pass through the origin. These deviations can be mitigated by variant pretension ratio among cables based on corresponding postures or the by introducing feedback loops. To further investigate the effect of the uniform pretension, Figure 8b demonstrates the deviation values at a pitch angle of $33^{\circ }$ (60% of workspace limit) with respect to different pretension ratios. Note that the distribution is too narrow to visualise the box. The whiskers are given by the oscillation occur at each steep change of the pretension ratio. The results generally exhibit a linear relationship for such pretension approach.

To evaluate the fault tolerance of the mechanism, we examined the consequences of broken cables by unbinding cables from the end effectors. For no more than two cable failures, whether the mechanism can still operate is listed in Table 1. A full failure only occurs in three cases which share a common pattern—the two faulty cables are opposite to each other. This causes absence of indispensable force pairs to restrain the mechanism in a stable state. For cases that are still operational, the performance of the mechanism is degraded to different levels correlated to combinations. With regard to three cable failure cases, we found the only pattern that it can still operate is that the cables are evenly separated, which are either $M_{1}N_{1}$–$M_{2}N_{2}$–$M_{3}N_{3}$ or $M_{1}N_{3}$–$M_{2}N_{1}$–$M_{3}N_{2}$. Such combination introduces non-eliminable roll motion but retains the functional pitch and yaw motion. Although the loss of cables results in smaller workspace and accuracy, the mechanism shows good performance overall in fault tolerance.

With a time step of 0.005 s, the simulation can run at about 46% of real time (no contact in the scene, CPU AMD Ryzen 2600X, GPU GTX1060, Ubuntu 20.04, single thread). The simulation speed is greatly affected by the total quantity of FEM elements in the scene. It can be improved with larger time steps to enable real-time interaction of the mechanism at a cost of losing some simulation details such as the reflection of oscillation.

### 3.3. Prototype Test Results

**Figure 9:**
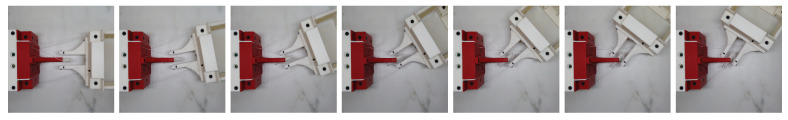
Prototype pitch motion snapshot from $0^{\circ }$ to $55^{\circ }$. From left to right the expected angles: $0^{\circ },10^{\circ },20^{\circ },30^{\circ },40^{\circ },50^{\circ },55^{\circ }$.

**Figure 10:**
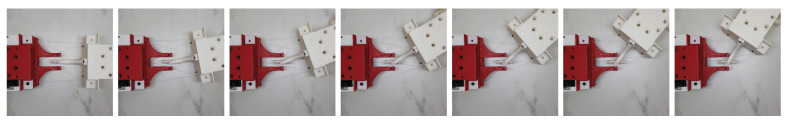
Prototype yaw motion snapshot from $0^{\circ }$ to $55^{\circ }$. From left to right the expected angles: $0^{\circ },10^{\circ },20^{\circ },30^{\circ },40^{\circ },50^{\circ },55^{\circ }$.

**Figure 11:**
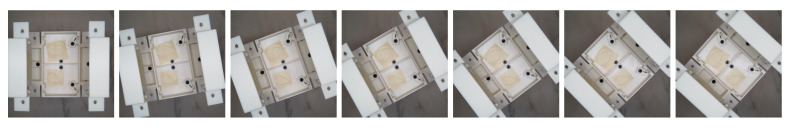
Prototype roll motion snapshot from $0^{\circ }$ to $60^{\circ }$. From left to right the expected angles: $0^{\circ },10^{\circ },20^{\circ },30^{\circ },40^{\circ },50^{\circ },60^{\circ }$.

The prototype test trials were conducted on each component motion independently. We firstly measured the deviation with a single run and then tested the repeatability. The pitch, yaw and roll motion sequences of the prototype are shown in Figure 9, Figure 10 and Figure 11 respectively.

The pitch and yaw motion measurements were taken from $0^{\circ }$ to $55^{\circ }$ with $5^{\circ }$ step size for both outbound and inbound direction. The results indicate the observed tilt angles exceed the expected values, which matches the simulation results. For pitch motion, a $55^{\circ }$ input gives an output of $56.88^{\circ }$, and $56.71^{\circ }$ for the yaw motion. The results also confirm the effect of pretension that provides more rigidity to the mechanism. For instance, there is approximately $1.3^{\circ }$ pitch angle difference between the loosed and pretensioned mechanism caused by gravity at reference posture.

The roll motion measurements range up to $60^{\circ }$ with the same step size. The results reveal that the maximum angle the prototype can reach is about $47.39^{\circ }$, where from $50^{\circ }$ to $60^{\circ }$ inputs the motion angle only increases by about $3.23^{\circ }$. This matches the estimation in simulation as the closer a vertex is to the roll extreme position, the higher is the tension force needed to compensate the impact of moment arm difference.

**Figure 12:**
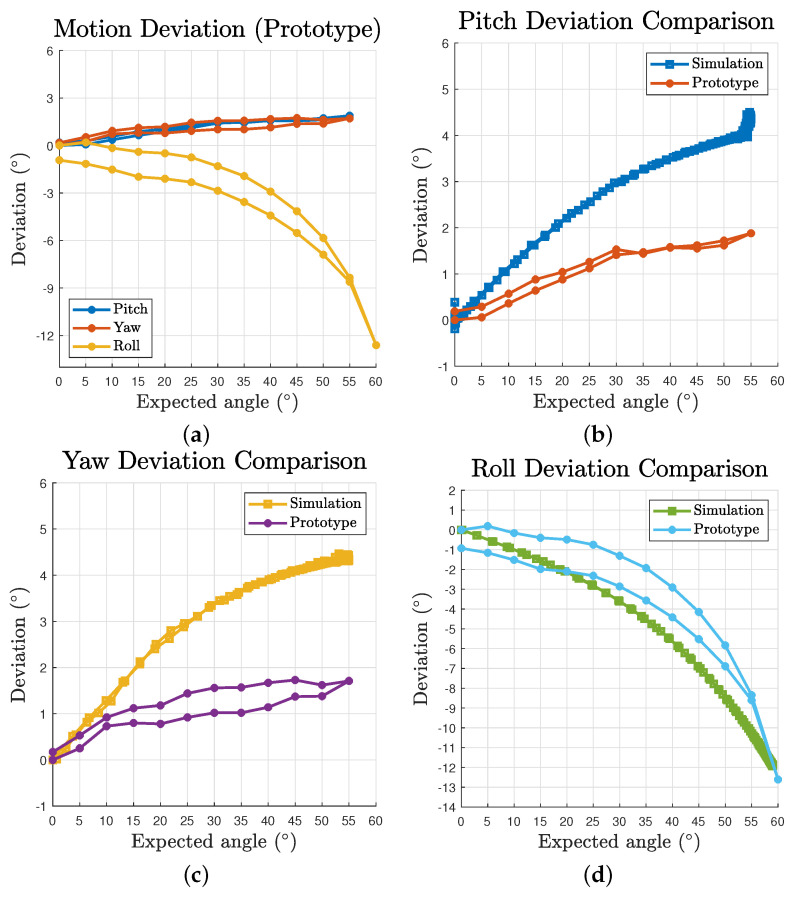
Prototype deviation and comparison with simulation results: (**a**) deviation with respect to expected angles for all three motions; and (**b**–**d**) comparison between simulation and prototype deviation for pitch, yaw and roll motion, respectively.

Based on the test results, the deviation are plotted in Figure 12a. The maximum deviations of pitch, yaw and roll are $1.88^{\circ }$, $1.71^{\circ }$ and $-12.61^{\circ }$, respectively. The data were offset with the intrinsic deviation at $0^{\circ }$ to evaluate the relative accuracy to the start status of the trials. The prototype results curve shares a similar pattern to that in the simulation (Figure 8a). However, a clear sign of hysteresis of outbound and inbound motion can be observed as a result of the property of practical cables. Figure 12b–d gives the comparison between the simulation and prototype. Although the overall pitch and yaw deviation of the prototype is approximately half the value of the simulation’s, which could be easily caused by errors introduced during assembly, it reveals a common trend of slowing down growth curve. As for the roll motion, the comparison exhibits a honest correlation between the simulation and prototype patterns. However several facts were also observed during the test. For yaw motion, due to a larger practical volume of the passive substructure, the actuated cables could collide with the struts for input angles greater than $45^{\circ }$. The prototype’s passive substructure is difficult to further reduce in size as it would be too small to be assembled or provide sufficient strength. For pitch motion, when the input angles are greater than $40^{\circ }$ for a downward motion, the mechanism would occasionally lose its equilibrium status as the cables could get pulled beyond the origin on the yz plane. We identified that is mainly caused by insufficient pretension of the passive substructure as there is obvious inward displacements after introducing pretension to the system, which eventually leads to reduced pretension ratio of the whole structure. In addition, the test results vary in a small range each time the prototype is disassembled and assembled, which is within expectation as a tensegrity and compliant structure.

The repeatability test of the mechanism took trials continuously between neutral and maximum postures for each component motion for 100 cycles. The measured deviation differences are listed in Table 2. The results demonstrate a good repeatability and consistency of the mechanism.

## 4. Conclusions and Future Work

In this paper, we present a versatile tensegrity mechanism made up of two separate substructures. The mechanism inherits biological tensegrity characteristics of light weight and compliance, while brings in improved agility, motion efficiency and compatibility adopting our design factors compared with existing tensegrity mechanisms. Unlike traditional Class-1 based tensegrity robots, our mechanism can be easily integrated with conventional robot systems to exploit advantages of both systems owing to its clean segmentation between the structural and mechanical parts and thus the impact on payload capacity due to introducing tensegrity is mitigated. The absolute motion extremes of the mechanism are analysed with its geometric model and the FCC algorithm for providing workspace reference during operation. Its $162^{\circ }$, $142^{\circ }$ and $118^{\circ }$ wide theoretical operation span expands the application scenarios and design potentiality. The simplicity of the model gives a straightforward inverse kinematics, reducing computation load at run-time. The accordant patterns of the simulation, which is the first physically accurate wheeled tensegrity robot simulation, and the prototype results validate that our mechanism is capable of operating reliably with designed properties in the compact design and is fault tolerant. The fusion of original tensegrity features and freshly introduced advantages makes the tensegrity mechanism ideal for planetary exploration and harsh environment robot applications. For instance, the mechanism can be applied to articulated rovers or snake-like robots as segment linkages that provide omnidirectional movement to facilitate exploration and natural terrain adaptation capability. It can also replace conventional ball-and-socket joints in biomimetic limbs such as the shoulder, elbow, hip and complex neck joints for humanoid robots, improving weight and flexibility while providing compliance for safer operation alongside humans. A waterproof version can be used for compliant articulated robotic fishes, enabling underwater vectored swimming thrust and multi-axis positioning. Our tensegrity mechanism enlarges the concept of how tensegrity robots can be designed to minimise common shortcomings and improve applicability. However, as a compliant system, the desired flexibility is greatly dependent on the implementation and environment of operation. Since the FCC indicates the mechanism has deformation tolerance boundaries when in active mode, it is crucial to determine appropriate cable properties and pretension ratios for predictable operation. In addition, it is challenging to achieve precise control of the mechanism compared to rigid robotic joints due to the extra displacements caused by its compliant nature and external factors, such as gravity, which is clearly noticeable in the test results. Another factor affecting the accuracy is the potential displacement of the passive substructure along its radial direction, where there are limited constraints at the neutral posture. Therefore, a key part of future work is to investigate approaches to reduce such inaccuracies. This could include introducing posture feedback to the mechanism, control logic based on dynamics of the system and probabilistic control methods that take into account uncertain environmental forces. In addition, eliminating the bending forces that exist in our present design will be investigated to further improve its structural efficiency.

## Figures and Tables

**Table 1:** Operation status of the robot: under no more than two cables failure condition; $N_{3}$ and $M_{2}$ are arranged in the vertical positive and negative direction, respectively, in our simulation.

Cable	$M_{1}N_{3}$	$M_{3}N_{3}$	$M_{1}N_{1}$	$M_{2}N_{1}$	$M_{3}N_{2}$	$M_{2}N_{2}$
$M_{1}N_{3}$	✓	✓	✓	✓	✓	×
$M_{3}N_{3}$	/	✓	✓	×	✓	✓
$M_{1}N_{1}$	/	/	✓	✓	×	✓
$M_{2}N_{1}$	/	/	/	✓	✓	✓
$M_{3}N_{2}$	/	/	/	/	✓	✓
$M_{2}N_{2}$	/	/	/	/	/	✓

**Table 2:** Difference of deviation at maximum expected angle: compared with the first cycle during repeatability test.

Motion	Pitch	Yaw	Roll
Half complete difference (${}^{\circ }$)	1.25	0.65	−0.77
Full complete difference (${}^{\circ }$)	1.42	0.52	−0.79

## Data Availability

Not applicable.
